# Phylogeographical Pattern and Population Evolution History of Indigenous *Elymus sibiricus* L. on Qinghai-Tibetan Plateau

**DOI:** 10.3389/fpls.2022.882601

**Published:** 2022-06-29

**Authors:** Mengli Han, Jingxue Zhang, Daxu Li, Shengnan Sun, Changbing Zhang, Chuanjie Zhang, Lijun Yan, Yongzhuo Guan, Lili Chen, Yuxia Guo, Minghong You, Wenlong Gou, Xinrui Li, Jiajun Yan, Shiqie Bai, Xuebing Yan

**Affiliations:** ^1^College of Animal Science and Technology, Yangzhou University, Yangzhou, China; ^2^Sichuan Academy of Grassland Sciences, Chengdu, China; ^3^College of Animal Science and Technology, Henan Agricultural University, Zhengzhou, China

**Keywords:** MaxEnt, diversity, differentiation, expansion, phylogeography, wheatgrass

## Abstract

Elymus sibiricus L. is a perennial allotetraploid belonging to Triticeae of Poaceae, *Elymus* L., as the type species of genus *Elymus* L. The existing geographical distribution pattern and genetic spatial structure of *E. sibiricus* on Qinghai–Tibetan Plateau (QTP) are not yet clear. In this study, population genetic structure and demography history of 216 individuals from 44 *E. sibiricus* populations on QTP were studied used specific-locus amplified fragment sequencing (SLAF-seq). The result of genetic diversity showed that there was no single genetic diversity center was observed across all *E. sibiricus* populations. The results of genetic variation showed that 44 populations were clearly divided into the following three groups: Qinghai Plateau (Group I), South Tibet (Group II), and Hengduan Mountains (Group III). From the three analyses of AMOVA, Mantel test and Treemix, strong genetic differentiation across all populations and low genetic differentiation among populations within three groups. Molecular dating indicated that *E. sibiricus* diverged at 16.08 Ma (during the early Miocene) can be linked to the Himalayan Motion stage of QTP uplift. It is speculated that the reasons affecting the current phylogeographical pattern are as follows: (1) The environmental changes due to the uplift of the QTP; (2) The geographic distance between the populations (Groups I and III are close in geographic distance, and gene flow are frequent); (3) Geographical barriers (the Tanggula and Bayangela Mountains between Groups I and II). This study provides new evidence and historical perspective to the future exploration of the evolution and geographic distribution pattern of *Elymus* L.

## Introduction

Understanding the underlying phylogeographical processes of the species distribution in some areas may help guide prioritization in conservation. It also may facilitate forecasts on the ecosystem services under the future climate conditions. It is believed that geological events and climate fluctuations have profoundly affected the distribution of species and the history of population dynamics in mountainous areas (Hewitt, 2004). The global climate change, especially the simultaneous climate that has fluctuated since the Quaternary Ice Age, has had a severe impact on the distribution of species, further forming plant differentiation and the current distribution pattern (Tian et al., 2010; Guo et al., 2012). In general, the response of species to Quaternary climate change includes changes in scope by forcing organisms into sanctuaries and re-colonizing under more favorable conditions (Taberlet et al., 1998; Hewitt, 2000). In the past 21–26 years, a large number of systematic geography studies in Europe and North America have summarized the evolution history of Quaternary flora in these regions (Abbott, 2000; Hewitt, 2004; Petit et al., 2005). Species conservation is an urgent global issue, especially in biodiversity hotspots, such as the QTP and the surrounding mountains, which are the highest priority areas for conservation research and implementation (Myers et al., 2000; Beger et al., 2015).

Qinghai–Tibetan Plateau is the highest plateau in the world covering an area of about 2.5 million km^2^ (Sun et al., 2014). It is one of the most important grazing land as well as natural reserve in China, which has harsh attributes and diverse ecological conditions, such as drought, low temperatures, strong winds, and high radiation (Yan et al., 2009). The QTP began to uplift 50 million years ago (Eocene), especially from Miocene to Pliocene (Miocene, 23–2.6 Ma), which greatly changed the surface pattern of the region. The rapid and extensive uplift of the QTP was considered a major driving force in shaping such high species diversity, and can generate geographic barriers between populations and restrict gene flow (Mao et al., 2010; Xu et al., 2010). Several cycles of climate change that produced mountain glaciers seem to have occurred in the Holocene (Zheng et al., 2002; Wang et al., 2012). This change of topography and geomorphology drives the genetic differentiation of plant related species and intra-specific populations. Climate change during the Quaternary Ice Age had a significant impact on the genetic distribution of plants on the QTP (Hewitt, 2000). The uplift, climate change of QTP, and restricted gene flow will lead to divergence between populations and new speciation over time due to the enhanced effects of genetic drift and/or natural selection (Hoskin et al., 2005). Phylogenetic, phylogeographic, and ecological studies support plant diversifications on the QTP through multiple mechanisms such as allopatric speciation *via* geographic isolation, climatic oscillations and divergences, pollinator-mediated isolation, diploid hybridization and introgression, and allopolyploidy. Nevertheless, a few recent studies examined the evolution of unique endemic plants and they have found that several monotypic plant genera in the alpine regions of the QTP are nested within larger genera (Friesen et al., 2000; Tian et al., 2011; Liu et al., 2012; Nie et al., 2013; Wen et al., 2014). Unfortunately, almost 90% of alpine grasslands on QTP region have been degraded due to overgrazing, rodent activities, and climate change in the recent years (Dong et al., 2013; Li et al., 2013). These factors can severely affect the feasibility of protecting *Elymus* L. germplasm resources. Much work especially on the widely distributed species with dominant composition and decisive function of grassland community is needed toward understanding the evolutionary mechanisms of plant diversifications on the QTP ecosystem.

With its diverse natural landscape and complex geological and climatic history, the QTP is a hotspot for biodiversity and a fascinating natural laboratory for studying how species have evolved and adapted (Li et al., 2012). *Elymus sibiricus* L. is the typical species of the genus *Elymus* L., which is the largest genus of Triticeae with about 150 species in temperate regions of the northern hemisphere (Barkworth and Dewey, 1984). *Elymus sibiricus* is a perennial, self-fertilizing grass, and an allotetraploid with the StStHH genome constitution, which is widely distributed on QTP (Zhou et al., 2016). It usually grows in wet meadows, sandy beaches by rivers, between open forests, on sunny or semi-shaded slopes of mountains, or valleys from 1,000 to 4,000 m (Ma et al., 2012). In addition to its native ecological function, *E. sibiricus* has become one of the most widely cultivated and used leading grass species on the QTP area in the recent years because of its high yield, good quality, and excellent adaptability to cold and arid climate. However, the related studies of *E. sibiricus* were mostly focused on ecological protection and genetic diversity by molecular markers, such as SSR (Simple Sequence Repeats) and AFLP(Amplified Fragment Length Polymorphism) (Xie et al., 2015; Zhou et al., 2016). Despite an increasing number of studies, the genetic divergence and phylogenomic analyses of the dominant community species, *E. sibiricus*, on the QTP mountain grassland remains poorly understood, which leads to the lack of scientific and targeted protection and utilization of the most important germplasm resources for grazing land.

In the recent years, many molecular markers have been used to infer the phylogeography and biogeography of species to understand their evolution patterns (Behura, 2010). Technological advances have made high-throughput sequencing a viable new strategy for studying population genetics (Hohenlohe et al., 2010). Specific-locus amplified fragment sequencing (SLAF-seq) is an efficient method of large-scale genotyping for genetic study, which is based on RRL (reduced representation library) and high-throughput sequencing (Sun et al., 2013). At present, SLAF-seq technology has been widely used in system evolution, population genetics, and adaptive evolution related to natural selection (Geng et al., 2016; Zeng et al., 2017). Consequently, in this study, the SNPs (single nucleotide polymorphism) in 216 individuals from 44 populations were explored using SLAF technology to (1) discover the distribution pattern of genetic diversity, spatial-temporal population genetic differentiation, and history of demography of *E. sibiricus* on the QTP; (2) speculate the population historical dynamics of *E. sibiricus*, and explore the historical reasons for the formation of the existing distribution pattern and possible sanctuaries, to clarify the effects of geological events such as uplift and glacial cover of the QTP and human activities on the distribution pattern of plant species diversity in this area; (3) provide a historical perspective for the phylogeographical formation of alpine meadow population structure and dynamics; (4) discuss the conservation strategy for improving collection, evaluation, and breeding efficiency and pertinence of *E. sibiricus* germplasm resources and also important for genetic evolution theory of polyploid populations and biodiversity conservation on the QTP. Consequently, the phylogeographical study of the dominant community species in mountain systems has motivated growing scientific interest in this research field.

## Materials and Methods

### Sampling Locality of *Elymus sibiricus*

**FIGURE 1:**
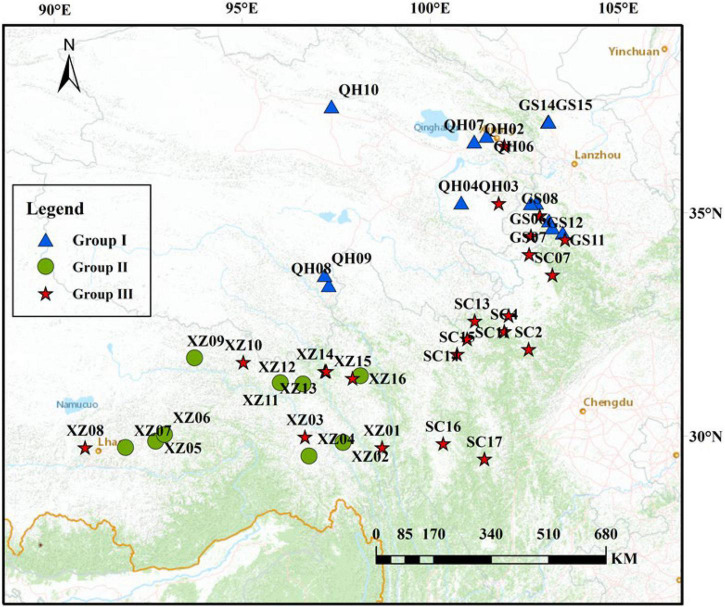
Geographic location of *E. sibiricus* populations in this study (the population geographic distribution of best results in clusters by Admixture).

A total of 216 individuals from 44 natural populations of *E. sibiricus* were collected from Gansu, Tibet, Sichuan, and Qinghai Provinces representing almost the entire distribution range of the species on QTP (Figure 1). The 44-specimen information about the sample id, sample number, latitude, longitude, and altitude was listed in Supplementary Table 1. The 44 populations have different geographic distribution. Five individuals were sampled for each population, and with individuals at least 30 m apart. Fresh leaves from each individual were collected and dried immediately in silica gel (Wang et al., 1996), and these individuals were stored in the Yangzhou University research lab for the next DNA attraction.

### The DNA Extraction and Sequencing

A total of 216 individuals genomic DNA was extracted from silica-dried leaf tissue using the DP350 Plant DNA Kit (Tiangen Biotech, Beijing, China). The concentration and purity of the DNA were assessed with ultra-micro photometer. Then the extracted DNA was stored at –20°C for further use.

According to the genomic size and GC content, the *E. sibiricus* genome (has not been published yet) was selected as the reference genome for predicting restriction enzyme sites. Finally, the optimal enzyme RsaI and EcoRV-HF was selected. Then, all DNA samples were digested separately, and a 474–504 bp restriction enzyme fragment length was defined as a SLAF tag. Rice has the characteristics of small genome; easy manipulation; and dense molecular markers, and has commonalities with other grass crops in gene sequence; structure; sequence and function, so it is hailed as a model species for genome analysis by the international scientific community (Upadhyaya, 2007). To evaluate the efficiency and quality of enzyme digestion, the *Oryza sativa* ssp. Japonica genome was selected as control group. After digestion, a single nucleotide (A) was added to the 30°C end using dATP at 37°C, and then dual-index adapters were ligated to the A-tailed DNA fragments. A PCR amplification was subsequently performed using diluted restriction-ligation DNA as the template. The E.Z.N.A.^®^ Cycle Pure Kit (Omega, London, United Kingdom) was used to purify the PCR products. The purified PCR products were pooled and incubated at 37°C with RsaI, T4 DNA ligase, ATP, and Solexa adapter. Then, the products were purified with a quick spin column (Qiagen, Hilden, Germany) and separated on a 2% agarose gel. All amplified fragments were diluted for the paired-end sequencing on an Illumina-HiSeq™ 2500 sequencing platform (Illumina, Inc., San Diago, CA, United States) at Beijing Biomarker Technologies Corporation (Beijing, China).

### SNP Calling

The raw sequence data were identified by dual-index sequencing, and the quality of the specimen sequence was evaluated after the adapter of the sequence data were filtered. The quality of sequencing (Q = –10 × log^e^_10_) and GC content were used to evaluate the single-base error rate of sequencing quality. Based on the sequence similarity, the high-quality paired-end reads were clustered using the BLAT software (Kent, 2002). More than 90% of the sequence similarity between individuals was identified as a SLAF locus (Sun et al., 2013). A total of 6,747,462 SLAF tags were predicted which were evenly distributed in the genome. All sequencing reads were aligned to the reference genome (*E. sibiricus* genome) by BWA software (Li and Durbin, 2010). The genome analysis GATK and SAMtools were used for SNP calling, and the resulting data were combined into a SNP data set (Mckenna et al., 2010). Eventually, the SNPs with a minor allele frequency (MAF) more than 5% and integrity more than 50% were selected. The raw data were submitted to the National Center for Biotechnology Information (NCBI) under the BioProject PRJNA811778.

### Population Genetic Analyses

A total of 30,159,737 SNPs was developed to construct the population structure and calculate genetic diversity. The measures of genetic diversity included the expected heterozygous number (He), Nei’s diversity index (H), observed allele number (Na), observed heterozygous number (Ho), Shannon’s wiener index (I), and polymorphism information content. These indexes can be calculated by POPGENE (Yeh et al., 1999). The spatial distribution map of genetic diversity was drawn by Arcmap software based on inverse distance weighting (IDW) (Mattioni et al., 2017). The regression analyses were conducted with genetic diversity indexes against altitude, longitude, and latitude to identify geographic patterns in the geographic pattern of genetic diversity. Analysis of molecular variance (AMOVA) was used to assess the degree of population differentiation. Additionally, pairwise fixation index (*F*_*ST*_) between populations was also computed the genetic differentiation. Analysis of molecular variance and *F*_*ST*_ were calculated by Arlequin (Excoffier et al., 2005). The SNPs were used calculate genetic distance among populations by the Kimura two-parameter model and the pairwise deletion option parameters.

The Phylogenetic tree was constructed by MEGA X program (Sudhir et al., 2018) with the following parameters: Neighbor-joining method and 10,000 bootstrap replicates. Based on the selected SNPs, the population structure was analyzed by Admixture software (Alexander et al., 2009). When the number of populations (K) was ranging from 1 to 10, clustering is carried out. The cross-validation error rate (ΔK) was used to determine K (minimum value). A principal component analysis (PCA) whose result can be used to verify with the results of other clustering methods and was calculated by EIGENSOFT (Price et al., 2006). To verify whether isolation-by-distance (IBD) exists among populations, Mantel test was used to examine for correlation between genetic distance and geographical distance in GenAlEx (Peakall and Smouse, 2010).

The linkage disequilibrium (LD) decay rate is crucial for maintenance of disequilibrium in a population (Pradhan et al., 2020). Because LD may affect both PCA and Admixture, the marker set was pruned by excluding SNPs in strong LD using PLINK software (Purcell et al., 2007). The linkage disequilibrium (LD) level was assessed using pair-wise LD as *r*^2^-value (Pearson’s squared correlation coefficient) by PLINK software (Purcell et al., 2007) based on the formula proposed by Hill and Robertson (1968). The *r*^2^-value was calculated between all pairs of SNPs with inter-SNP distances of less than 10 kb (*r*^2^ and LD-window parameters).

### Demography History

To infer the split and Admixture history among groups of 44 populations, the TreeMix (Pickrell et al., 2012) was used based on the SNPs. TreeMix, which simultaneously infers a tree of relationships and “migration events,” used allele frequency data to approximate an unrooted maximum likelihood (Beger et al., 2015) population tree. The effect of migration on the residual covariance matrix was examined by the stepwise likelihood method, and the optimal location of migration events in the population tree was determined. There are few reports of fossil data of *E. sibiricus*; therefore, based on the data of *Elymus* species and *Aegilops tauschii*^1^, the MCMCtree program implemented in the PAML v4.8 package (Yang, 2007) was used to estimate the divergence times among the species using a maximum likelihood method (Beger et al., 2015). The independent rates model (clock = 2), which assumes uncorrelated relaxed molecular clock. It was used to specify the prior of rates among the internal nodes, which followed a log-normal distribution. The three parameters (birth rate, *l*; death rate, *m*; and sampling fraction, *r*) in the birth–death process with species sampling were specified as 1, 1, and 0, respectively. A loose maximum bound for the root was set between 3 Ma and 9.4 Ma. The first 50,000 cycles in MCMCtree were discarded as burn-in, and every 50 cycles were sampled to obtain a total of 100,000 individuals.

To make sense of the population dynamic expansion history, the DNASP was used to calculate the mismatch distribution (Rozas et al., 2003). It is supposed that multimodal and rough distributions would characterize populations that have been stable for a long time, whereas populations that have experienced a sudden demographic expansion should display a unimodal and smooth distribution (Rogers and Harpending, 1992). Meanwhile, the neutrality test statistics was carried for population genetic data. Tajima’s *D* (Tajima, 1989), Fu and Li’s *D*, Fu and Li’s *F* (Li and Fu, 1993) statistics were used to estimate the population expansion and direct selection that the population underwent during the evolution process and the result can be complete by DNASP software (Rozas et al., 2003). These statistics are sensitive to the past population expansion and contraction. It is generally expected to be negative in the case of population expansion, and positive in the case of population contraction or the recent bottleneck events (Tajima, 1989; Li and Fu, 1993). To infer that *E. sibiricus* experienced bottleneck effect in the recent years, the SMC++ software was used to explore population size changes over time (Terhorst et al., 2016).

### Analysis of Ecological Niche Model

The maximum entropy modeling (MaxEnt) (Phillips et al., 2006) and ArcGIS were used to construct the suitable area distribution of *E. sibiricus* during the Last Inter-Glacial period (LIG ∼120,000–140,000 years ago), Last Glacial Maximum (LGM ∼22,000 years ago), Mid-Holocene (6,000 years ago), present (∼1950–2000), and projected future (2070) (Phillips et al., 2006). The species distribution data included 88 sites come from field sampling and the Global Biodiversity Information Facility (GBIF)^2^. Environmental data (Supplementary Table 2) was downloaded from the WorldClim database^3^ and the map of QTP (1:400 million) from the National Geographic Center of China. The receiver operating characteristic (ROC) evaluated the accuracy of the model by the area under the curve (AUC) (Ritchie et al., 2017). The evaluation criteria of AUC (Greiner et al., 2000) was liated as follows: Less accurate (0.5 < AUC ≤ 0.7), moderately accurate (0.7 < AUC ≤ 0.9), highly accurate (0.9 < AUC < 1), and perfect tests (AUC = 1). In addition, the key environmental factors were selected by Jackknife method (Peterson and Cohoon, 1999). Finally, we used the natural discontinuity method to divide the suitable area of *E. sibiricus* into the following four grades: Not suitable, low suitable, moderately suitable, and highly suitable.

## Results

### Sequencing and SNP Calling

A total of 1,221.47 Mb reads were obtained from the Illumina HiSeq 2500 platform. The reads with a length of 125 bp were selected for the further evaluation and analysis to ensure the quality of the data. The average Q30 was 93.13% and the average GC content was 45.91%, which indicated that the reliability of the data. In addition, a total of 6,747,462 SLAF tags were explored, which 3,890,215 were polymorphic and the average depth was 5.10x. Finally, 30,159,737 SNPs were obtained in total (Supplementary Table 3).

### Genetic Diversity

**FIGURE 2:**
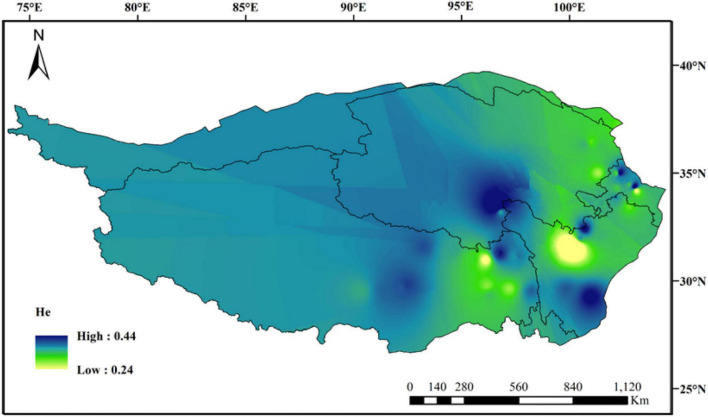
Genetic diversity map of 44 *E. sibiricus* populations: IDW interpolation of expected heterozygosity values (He).

The average values of observed heterozygous (Ho) and expected heterozygous (He) were 0.4167 and 0.3750, respectively. The maximum and minimum values of Nei’s diversity index (H), Shannon–Wiener index (I), and polymorphism information content (PIC) appeared correspondingly in population QH09 and SC15, and these average values at species level were 0.4196, 0.5543, and 0.2971, respectively (Table 1). Additionally, the regression analysis showed that there was a significant decline in Ho with increasing longitude (Supplementary Figure 1A: *R*^2^ = 0.314, *p* < 0.001) and latitude (Supplementary Figure 1B: *R*^2^ = 0.158, *p* = 0.008), and a significant ascent in Ho with increasing altitude (Supplementary Figure 1C: *R*^2^ = 0.22, *p* = 0.001). However, there were no significant differences in other genetic diversity indexes except Ho. The spatial distribution of genetic diversity of *E. sibiricus* across the whole sampling range was shown as that genetic diversity values were lower in northeastern region and western Hengduan Mountains of QTP and higher in the center and southeast of QTP (Figure 2). However, from the whole QTP, multiple genetic diversity centers were obviously observed across all *E. sibiricus* populations.

**TABLE 1:** Intra-population genetic diversity of 44 *E. sibiricus* populations.

Population	*Na*	*Ne*	*Ho*	*He*	*H*	*I*	*PIC*
QH02	2	1.6468	0.3191	0.3687	0.4105	0.5466	0.2925
QH03	2	1.5767	0.2095	0.3463	0.3878	0.5234	0.2799
QH04	2	1.6394	0.4942	0.3657	0.4085	0.5431	0.2905
QH06	2	1.6163	0.4590	0.3567	0.3982	0.5331	0.2848
QH07	2	1.6570	0.5132	0.3734	0.4162	0.5522	0.2958
QH08	2	1.6706	0.5272	0.3789	0.4222	0.5585	0.2994
QH09	2	1.8133	0.3201	0.4412	0.5042	0.6307	0.3413
QH10	2	1.6639	0.5529	0.3748	0.4186	0.5533	0.2963
GS02	2	1.7942	0.2196	0.4298	0.4801	0.6169	0.3330
GS03	2	1.6033	0.2766	0.3551	0.3964	0.5328	0.2851
GS06	2	1.6137	0.2108	0.3647	0.4074	0.5457	0.2932
GS07	2	1.6733	0.1782	0.3835	0.4291	0.5650	0.3035
GS08	2	1.6810	0.4614	0.3858	0.4312	0.5673	0.3047
GS09	2	1.6743	0.4180	0.3855	0.4332	0.5679	0.3054
GS10	2	1.6293	0.4303	0.3656	0.4095	0.5446	0.2918
GS11	2	1.8155	0.1273	0.4397	0.4921	0.6284	0.3397
GS12	2	1.4780	0.0906	0.3156	0.3524	0.4922	0.2632
GS14	2	1.6595	0.4198	0.3780	0.4238	0.5588	0.3000
GS15	2	1.5225	0.3090	0.3206	0.3591	0.4926	0.2619
SC02	2	1.6345	0.2464	0.3694	0.4119	0.5497	0.2950
SC04	2	1.6399	0.2395	0.3733	0.4165	0.5547	0.2980
SC07	2	1.6028	0.3839	0.3553	0.3961	0.5331	0.2853
SC11	2	1.6594	0.5730	0.3700	0.4124	0.5468	0.2921
SC13	2	1.7549	0.3743	0.4137	0.4617	0.5984	0.3223
SC14	2	1.6922	0.5918	0.3845	0.4289	0.5638	0.3020
SC15	2	1.3461	0.2288	0.2382	0.2656	0.3943	0.2042
SC16	2	1.7061	0.5559	0.3930	0.4396	0.5745	0.3085
SC17	2	1.7518	0.6390	0.4118	0.4706	0.5963	0.3210
XZ01	2	1.7031	0.3183	0.3914	0.4373	0.5725	0.3073
XZ02	2	1.6053	0.4796	0.3489	0.3900	0.5229	0.2786
XZ03	2	1.6024	0.4400	0.3509	0.3921	0.5264	0.2810
XZ04	2	1.6526	0.5097	0.3696	0.4133	0.5472	0.2926
XZ05	2	1.7130	0.5838	0.3933	0.4388	0.5739	0.3079
XZ06	2	1.7224	0.5789	0.3984	0.4448	0.5801	0.3116
XZ07	2	1.7059	0.5650	0.3914	0.4370	0.5722	0.3070
XZ08	2	1.6700	0.5432	0.3763	0.4204	0.5547	0.2969
XZ09	2	1.7001	0.5057	0.3947	0.4511	0.5782	0.3111
XZ10	2	1.6776	0.5756	0.3782	0.4217	0.5564	0.2977
XZ11	2	1.6340	0.4227	0.3625	0.4046	0.5392	0.2882
XZ12	2	1.5513	0.4871	0.3230	0.3597	0.4919	0.2604
XZ13	2	1.7061	0.5838	0.3944	0.4508	0.5770	0.3101
XZ14	2	1.7616	0.273	0.4161	0.4638	0.6009	0.3237
XZ15	2	1.6857	0.5871	0.3826	0.4266	0.5618	0.3010
XZ16	2	1.7014	0.5062	0.3884	0.4330	0.5683	0.3046
All populations	2	1.6593	0.4166	0.3750	0.4196	0.5543	0.2971

### Genetic Differentiation and Spatial Structure Among Populations

**FIGURE 3:**
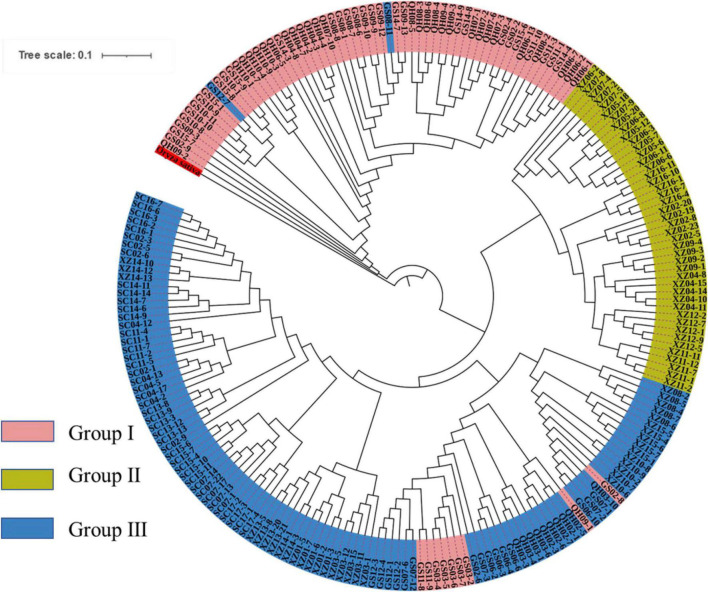
Phylogenetic tree constructed with polymorphic SNPs between three groups.

**FIGURE 4:**
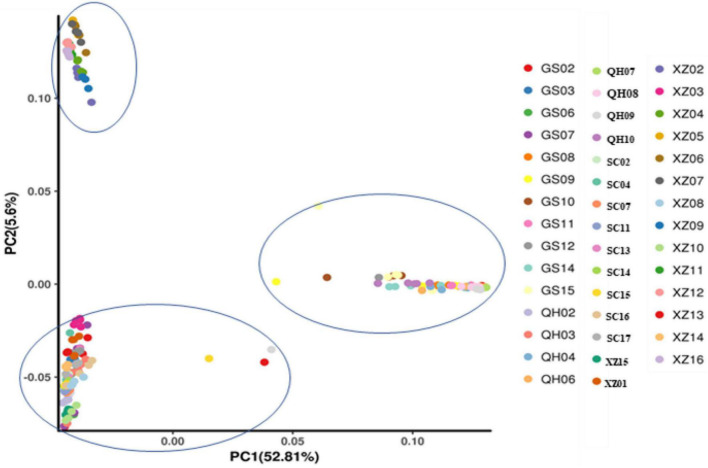
Principal components analysis of 44 *E. sibiricus* populations. Each circle represents an individual, and the circle color represents the population attribute of each individual.

To explore the divergence among populations, in this study, 216 individuals of 44 *E. sibiricus* populations were divided into three clusters based on the NJ tree as indicated with different colors in Figure 3. Notably, PCA further proved the discovery (Figure 4). Based on the population allele frequency, the PC1 and PC2 accounted for 52.81 and 5.6% of total variation respectively. Consequently, it was reasonable for us to divide 44 populations into three groups (Figure 1). Group I was located in the northeastern QTP, which contained 13 populations. Group II was located in South-Tibet river basin, which included nine populations mainly from the XZ region. Group III was located in Hengduan Mountains, which covered a wider range of individuals including 22 populations of the QH, GS, XZ, and SC regions.

Analyses of molecular variance of 44 *E. sibiricus* populations indicated that most of total variations mainly existed among groups and populations with the differentiation coefficient of 66.39 and 64.44%, respectively (Table 2). The AMOVA analysis against each group further was conducted. It is interestingly observed that the low inter-population genetic differentiation among populations within three groups, and the fixation index (*F*_*ST*_) of the three groups were 0.12, 0.397, and 0.453, respectively. It indicated the notable local gene flow among the populations within each group. In addition, the *F*_*ST*_ value among three groups (Table 3) showed Groups I and II > Groups I and III > Groups II and III. The Mantel test showed a significant positive relationship between *F*_*ST*_ and pairwise geographic distance, indicating a significant pattern of isolation by distance across all 44 *E. sibiricus* populations (Supplementary Figure 2; *r* = 0.307, *p* < 0.001).

**TABLE 2:** Results of hierarchical AMOVA for 44 populations of *E. sibiricus* sampled within four regions.

Grouping	Degree of freedom (Df)	Source of variation (SOV)	Variance Components (VC)	Percentage of Variation PV (%)	Fixation index (*F*_*ST*_)
All groups	2	Among groups	818.96623	66.39	0.664
	213	Among individuals within groups	54.96175	4.46	
	216	Within individuals	359.72917	29.16	
Group I	12	Among populations	194.91873	12.03	0.120
	64	Within populations	1425.19363	87.96	
Group II	8	Among populations	334.12506	39.66	0.397
	44	Within populations	508.36464	60.34	
Group III	21	Among populations	242.65029	45.3	0.453
	108	Within populations	293.04897	54.71	
All populations	43	Among populations	90.84763	64.44	0.644
	216	Within populations	50.1264	35.56	

**TABLE 3:** The *F*_*ST*_ value between three groups.

Groups	Group I	Group II	Group III
Group I	0		
Group II	0.72	0	
Group III	0.68	0.46	0

**FIGURE 5:**
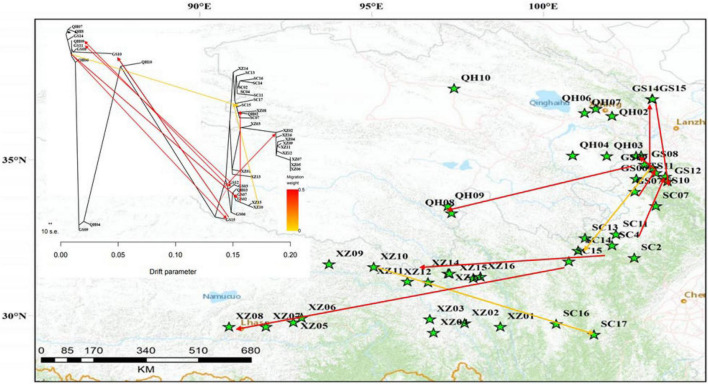
Phylogenetic network of the genetic relationships among the *E. sibiricus* populations with different possible migration events (*m* = 10, six migration events) inferred from TreeMix analysis. The graph shows the topology and branch lengths according to the drift parameter. Migration arrows were colored according to their weight. The scale bar shows 10 times the average standard error (s.e.) of the entries in the sample covariance matrix W.

**FIGURE 6:**
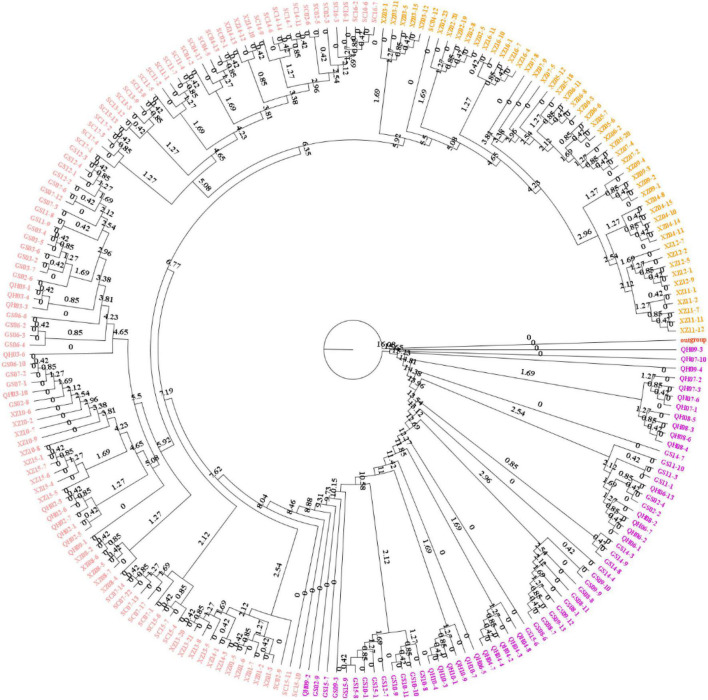
Phylogenetic tree constructed with polymorphic SNPs and divergence time scale between three groups.

From the unrooted maximum likelihood tree inferred by TreeMix (*m* = 10, adding six migration events, Figure 5), the low weight of these proposed migration events was related to the low proportion of alleles in the offspring from the ancestral populations. This result may reflect the past gene flow. Notably, a small scale of gene flow occurred among *E. sibiricus* populations throughout the QTP. The TreeMix tree showed the gene flow mainly existed between Groups I and III. There were limited gene flow between Groups II and III, and no gene flow between Groups I and II. Additionally, the divergence time among 216 *E. sibiricus* individuals on the ML tree (Figure 6) showed that Group I diverged earliest compared with the other two groups, and the divergence time was 8.8 Ma between Groups I and II (With *Aegilops tauschii* as an outgroup, the divergence time between them was about 16.5 Ma). Whereas the late divergence between Groups II and III happened 5.92 Ma ago. Thus, it was probably to infer that the northeast of QTP was the origin area of *E. sibiricus* population 16.08 Ma ago.

**FIGURE 7:**
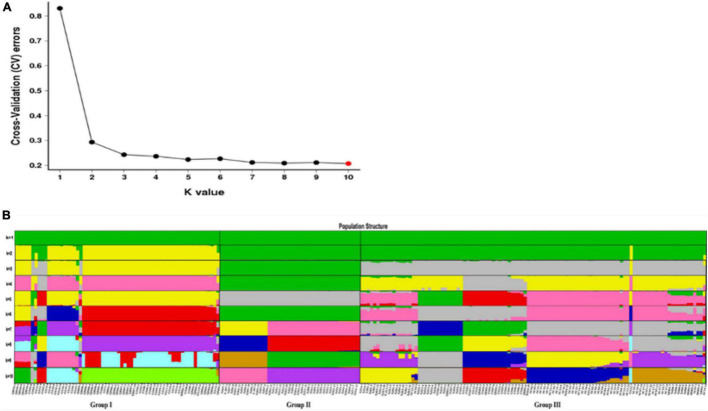
**(A)** Admixture estimation of the number of groups for *K* values ranging from 1 to 10. **(B)** Population structure analysis of the 216 individuals based on 30,159,737 SNPs. The bars in the *x*-axis indicate different individuals. Colors in each row represent structural components.

The population structure was analyzed using the Admixture software to further understand the degree of Admixture in the populations. As shown in Figure 7A, the best *k*-value was 10. However, when *k* = 3, CV errors showed a significant decline, and CV errors value was already very low. So, the best *k*-value can be chosen as 2 or 3, indicating a strong genetic cluster among populations. When *K* = 2, a total of 216 individuals were divided into two clusters and Group I was separated to other groups. When *K* = 3, the obvious grouping (Groups I, II, and III) was observed, and the cluster result was consistent with PCA and phylogenetic relationship. The result indicated that *E. sibiricus* populations did not originate independently from one location on the QTP. All individuals with a low degree of Admixture were seen from all studied populations. From the dynamics of *K* = 2–10 (Figure 7B), Group I showed the evidence of some Admixture which may be due to gene flow and the recent expansion events. Groups II and III was stable and almost not admixed.

### Linkage Disequilibrium

**FIGURE 8:**
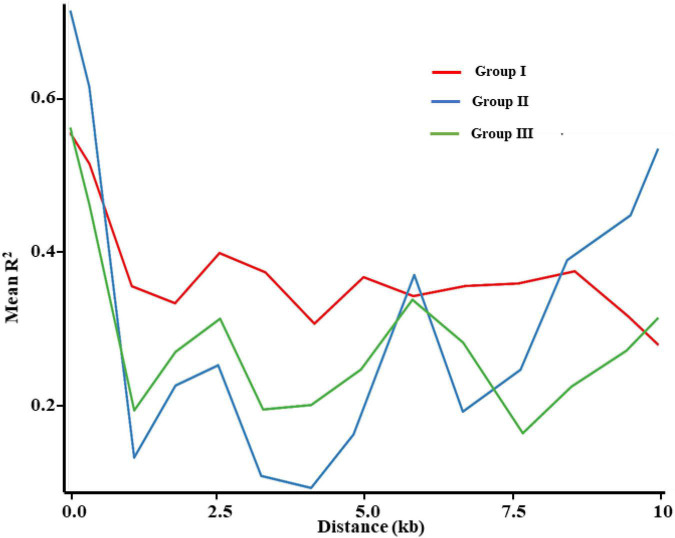
Decay of average linkage disequilibrium (LD) over distance among SNPs in *E. sibiricus* populations.

The decay of LD (*r*^2^) was calculated for the three groups and the maximum *r*^2^ values of the three groups were 0.5584, 0.7174, and 0.5646, respectively (Figure 8). The degree of LD decreased with the increasing marker distance. When the mean value of *r*^2^ declined to half from the initial value, the average distances of Groups I, II, and III were 10, 0.8121, and 0.9302 kb, respectively. The LD in Group I presented a less dramatic drop than in the two other groups, whereas LD extent was very similar between Groups II and III. This linkage disequilibrium decay pattern supported the results of the stronger bottleneck in Group I and relatively lighter selection pressure in Groups II and III.

### Demography Statistics

**FIGURE 9:**
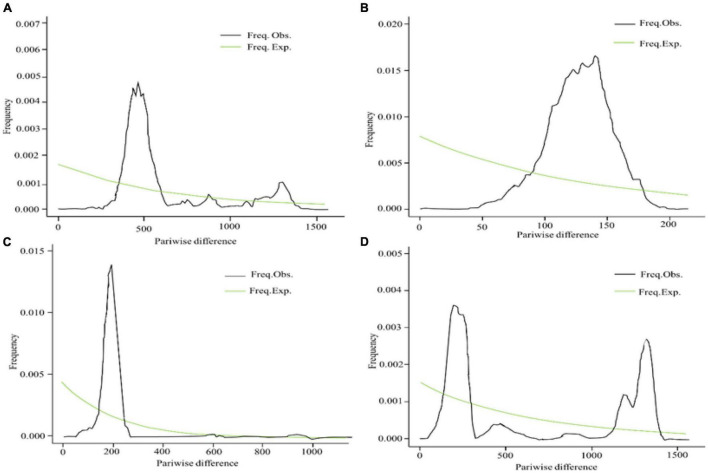
Mismatch distribution analysis for SNPs data of Group I **(A)**, Group II **(B)**, Group III **(C)**, and overall **(D)**.

**FIGURE 10:**
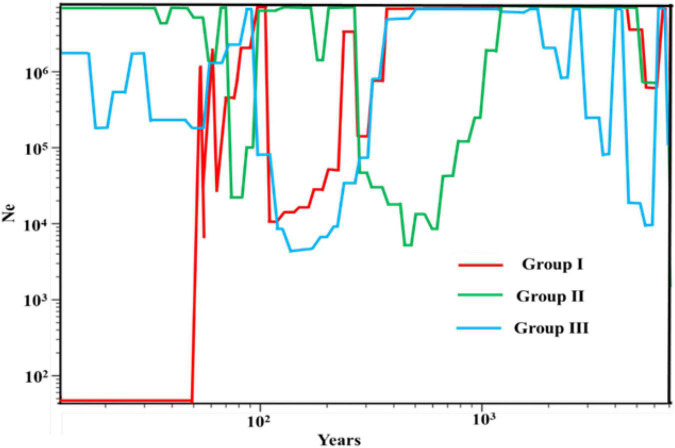
Results of effective population size inference for three groups.

To understand the demographic history of *E. sibiricus*, Tajima’s *D*, Fu, and Li’s *D*, Fu and Li’s F statistics were calculated to test for evidence of range expansion (Table 4). In all populations across the QTP, Fu and Li’s *D*, Fu and Li’s *F*-values were all significantly positive, indicating that the *E. sibiricus* possibly experience the obvious population contraction on the QTP. Likewise in Group I, significantly positive Fu and Li’s *D*, Fu and Li’s *F*-values suggested that these populations experienced the obvious contraction. In contrast, Tajima’s *D*, Fu and Li’s *D*, Fu and Li’s *F*-values for Groups II and III were all not significant, so it can infer that Groups II and III possibly did not experience the population expansion in the recent. On the other hand, the mismatch distribution for all populations showed a bimodal curve (Figure 9), which indicated the 44 populations of *E. sibiricus* on the QTP did not experience a large-scale population expansion. In addition, a bimodal curve of the mismatch distribution in Group I also indicated there no obvious population expansion. However, the unimodal Poisson distribution in Groups II and III suggested that they experienced one large-scale expansion recently. Therefore, the results of the comprehensive neutral test and the mismatch distribution suggested that Groups II and III possibly experienced populations expansion recently, but Group I as well as the whole *E. sibiricus* populations on the QTP likely experienced slight contraction. The results of demographic inference indicated over the last 10,000 years, the whole QTP *E. sibiricus* populations experienced decline trend (Figure 10). Groups I and II had similar population size between 10,000 and 1,000 years ago. Groups I and III had similar population size between 1,000 and 100 years ago. Starting from around 500 years ago, Group I experienced a decline which persisted until about 100 years ago, but about 70 years ago, Group I declined sharply. About 10,000 years ago, Group II experienced decline until about 600 years ago, the population size reached at 10^7^ to those day. Starting from about 500 years ago, Group III experienced decline until about 100 years ago, and then experienced a rapid expansion and slight population fluctuations. Eventually, the population size reached at about 10^6^. In addition, the populations of the three groups have expanded from 10,000 to 6,000 years ago (Figure 10), consistent with the MaxEnt events exactly at the period of LGM-Mid-Holocene transition.

**TABLE 4:** Neutrality tests of E. sibiricus populations based on SNPs data.

Grouping	Neutrality Tests		
	Tajima’s *D* (*p*)	Fu and Li’s *D* (*p*)	Fu and Li’s *F* (*p*)
Overall	3.01697	3.98901	3.99543
	*p* < 0.01**	*p* < 0.02*	*p* < 0.02*
Group I	1.91469	2.45317	2.63258
	0.1 > *p* > 0.05	*p* < 0.02*	*p* < 0.02*
Group II	0.86972	–1.95781	–0.8894
	*p* > 0.10	0.10 > *p* > 0.05	*p* > 0.10
Group III	–1.15319	–0.05842	–0.7314
	*p* > 0.10	*p* > 0.10	*p* > 0.10

*Significant; ** extremely significant.

### Potential Distribution

**FIGURE 11:**
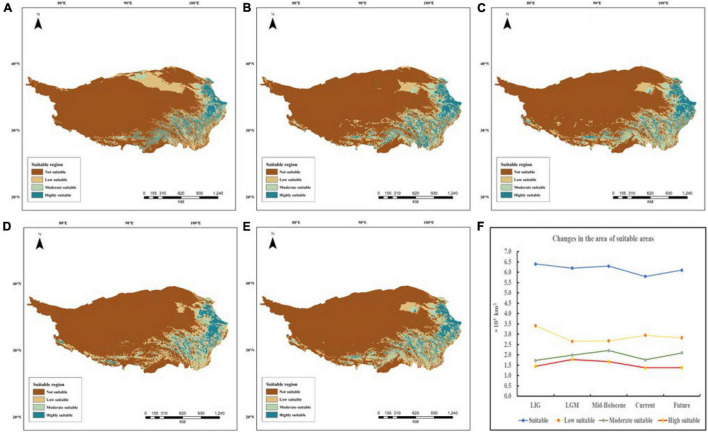
Suitable distribution maps of *E. sibiricus* during different periods on Qinghai–Tibet Plateau **(A)** the Last Inter Glacial period (LIG ∼120,000–140,000 years ago); **(B)** Last Glacial Maximum (LGM ∼22,000 ya); **(C)** Mid-Holocene (6,000 years ago); **(D)** present (∼1950–2000); **(E)** projected future (2070); **(F)** changes of suitable areas at different levels in five periods.

In this study, we modeled the LIG, LGM, Mid-Holocene, current, and the future distributions for *E. sibiricus* using MaxEnt version 3.4.1 as shown in Figure 11. MaxEnt model had high predictive ability and five periods AUC were acquired (LIG: AUC = 0.942, LGM: AUC = 0.933, Mid-Holocene: AUC = 0.931, current: AUC = 0.944, future: AUC = 0.938), which demonstrated the models had good model performances. According to the MaxEnt result, *E. sibiricus* distributed mainly at the northeast and southeast edge of QTP during the LIG period. During the LGM period, highly suitable area underwent a slight expansion, but the whole distribution persisted a slight contraction. The highly suitable area had a slight movement toward to the edge of QTP (Hengduan Mountains region). Populations exhibited expansion from the LGM to Mid-Holocene period. However, the highly suitable area had a slight contraction. Currently, potential suitable distribution further contracted in overall QTP range. Considering the changes of the potential distribution range in five periods, *E. sibiricus* had undergone the repeated historical process of contraction and expansion. Interestingly in the future, the species will potentially experience an obvious expansion. In addition, it was also found that the key environmental factors affecting the distribution of *E. sibiricus* were mean annual temperature (Bio1), mean temperature of coldest quarter (Bio11), annual precipitation (Bio12), and elevation (Elev) (Supplementary Figure 3).

## Discussion

### Genetic Diversity and Population Structure

Genetic diversity is the basis of species diversity and an important precursor to the study of any species, because the quantity and distribution of genetic diversity affect the evolution and reproductive potential of species or populations (Futuyma and Fairbairn, 1979). There is no obvious regular pattern of genetic diversity across all QTP *E. sibiricus* populations in this study. The QTP is characteristic of complex geographical environment, which may be the cause of variation in within-population genetic diversity. Mountain barriers may play a key role in species formation and diversity as their topographic complexity may lead to ecological stratification and environmental heterogeneity (Fjelds et al., 2012). The wide geographical distribution of *E. sibiricus* may also be one of the reasons for the difference in genetic diversity (Xie et al., 2015). The work of Hamrick and Godt (1996) has shown that geographic ranges were significantly correlated with plant variation at both species and population levels. The wide-ranging species maintain more variability than narrow ranging species. This finding will cause the difficult strategy for conservation and utilization of *E. sibiricus* germplasm, and prove QTP as its resourceful deposit. However, the spatial distribution of genetic diversity of *E. sibiricus* within the whole sampling range was shown that higher genetic diversity in the central and southeast of QTP (Figure 2). The result also found several populations had higher genetic diversity, speculating that these places may be the possible resourceful refuges. Shelter populations have a higher level of genetic diversity because they have a longer history of population dynamics as compared to post-glacial populations (Comes and Kadereit, 1998; Taberlet et al., 1998), which can be mirrored by the genetic diversity pattern in this study. In respect of the correlation between genetic diversity and altitude, the result of regression analysis shown that there was no significant correlation (Supplementary Figure 1). However, there were no significant differences in other genetic diversity indexes except Ho. Yan et al. (2009) indicated that a higher genetic diversity was observed in populations occurring at the medium altitudes (3,200–3,600 m) than those at the low and high altitudes for the two *Elymus* species (*Elymus nutans* and *Elymus burchan-buddae*) on the QTP. However, the results of our study were not completely consistent with their conclusion even in different *Elymus* species. The reasons for this difference were likely to be that the complex geological environment of a large-scale sampling area conceals the influence of altitude on genetic diversity, or the sampling sites higher than 3,800 m and lower than 3,000 m in this study are relatively scaring, and without covering the entire altitude range. Genetic structure analysis at the intra-specific level is important for future adaptive change or evolution (Schaal et al., 1991), which is informative for germplasm conservation. A PCA analysis, phylogenetic tree analysis, and Admixture analysis demonstrated that *E. sibiricus* showed a certain regional cluster (Figures 3, 4, 7). Populations from the same or similar geographical origin tend to cluster together, and 44 populations was divided into three genetic groups. These three groups were located in three separate locations with obvious geographical differences on the QTP. These findings will make it relatively easy for conservation and utilization of the wheatgrass germplasm on the QTP.

### Genetic Differentiation Among Populations and Groups

According to AMOVA analysis of all populations, the results of this study were consistent with previous genetic studies of *E. sibiricus*, which found that most of the variations were distributed within populations or geographical areas (Ma et al., 2008; Yan et al., 2010). Genetic variation and gene flow among populations are important factors to explain population structure and dynamics history (Behura, 2010). Analyses of the genetic differentiation of *E. sibiricus* populations showed obvious evidence of major geographical partitions and barriers to historical gene flow. A significant negative relationship between *F*_*ST*_ and pairwise geographic distance across all 44 *E. sibiricus* populations (Supplementary Figure 2: *r* = 0.307, *p* < 0.001), indicating that the isolation-by-distance model seems to describe the pattern of differentiation among the populations in this study. There was a significant correlation between genetic distance and geographical distance of *E. sibiricus* populations due to the main self-pollination breeding system of *E. sibiricus*. In general, selfing species commonly have lower levels of genetic diversity and higher differentiation between populations than outcrossing plants (Schoen and Brown, 1991; Hamrick and Godt, 1996). In addition to its self-pollination system, some geographical isolation *via* barriers, such as rivers and mountains, can prevent *E. sibiricus* from spreading to a very large scale geographical level. Bockelmann et al. (2010) hold that within a 100 m habitat range, *E. athericus* populations with unique habitats have undergone significant genetic variation. When the distance is greater than 60 km, the impact of geographic distance isolation becomes weaker, which confirms the impact of habitat conditions on the genetic variation of *Elymus* genus plants. Genetic differentiation among intra-specific populations was promoted by geographical barriers of a series of high mountains on the QTP, while the formation of mountain systems, river diversions and “Sky island” restricted intra-specific gene flow, disrupting genetic patterns and speciation (Matuszak et al., 2016). Therefore, the self-pollination, geographic barriers, and habitat effect may have led to current general population structure of *E. sibiricus* on the QTP. In addition, a low genetic differentiation was observed among populations within each of the three groups, suggesting that local gene flow existed three groups. Local gene flow not only caused by self-pollination system but also by extra power, such as grazing activity (Yan et al., 2010). In addition, the study also found the gene flow in the whole region seemed to be restricted by high mountains on the map (Table 2 and Figure 5). The higher within-group variation of the three geographical groups (66.39%) may be attributed to isolation by environment of sampling at a larger spatial scale (Nybom, 2004).

The uplift of the mountain triggered the differentiation of species, changed the genetic structure of alpine plants, and affected the evolution of alpine plants (Liu et al., 2012; Wen et al., 2014). The reasons for this genetic structure were geographic isolation or adverse environmental changes (Avise et al., 1987). To display the fine spatial differentiation pattern of *E. sibiricus*, three genetic groups was divided by the PCA, Admixture analysis and phylogenetic tree. Based on the topography of the QTP, it was possible that the Tanggula Mountains and Tongtian rivers formed a geographical barrier between the two groups (Groups I and II). Group II was distributed in the South-Tibetan river basin, which had almost no gene flow compared with the other two groups. The degree of Groups II and III was low with the cause of the river obstacle, and there were no gene flow among Groups I and II with the geographical isolation of Tanggula Mountains. Interestingly, the gene flow mainly existed between Groups I and III because two groups were not isolated by very huge physical barriers (Figure 5). In addition, due to the complex topography of the QTP, the separation of the high mountains led to the isolation from populations of each other, hindering the long distance spread of seeds and pollen, further promoting genetic differentiation among populations. By the *F*ST results inferred that the Tanggula and Bayan Har Mountains as the geographical barriers of the three groups, probably imposed divergence of them. Therefore, it can conclude that QTP specific mountainous environment will play an important role in shaping the genetic structure of *E. sibiricus* by preventing the gene flow. In summary, it is speculated that the reasons affecting the current phylogeographical pattern were as follows: (1) The environment changes due to the uplift of the QTP; (2) The geographic distance between the populations (Groups I and III are close in geographic distance, and gene flow are frequent); (3) Geographical barriers (the Tanggula and Bayan Har Mountains between Groups I and II).

### Species Divergence and Demographic History

In this study, the divergence time of the 44 *E. sibiricus* populations showed Group I in the northeast of QTP first split compare with other groups, and the divergence time was 16.08 Ma. The divergence time between Group III and Group II was 5.92 Ma (late Miocene-early pliocene). The lasting climatic aridification and other events following the Himalayan Motion during 12∼6 Ma possibly played a direct role in the diversification of Group II and Group III. In addition, the abiotic event during 16∼5.92 Ma that led to the differentiation of these groups may be QTP uplift in 13∼7 Ma (Harrison et al., 1992; Willis and McElwain, 2014). During the uplift of the QTP and adjacent Himalayan mountains, long-term geological events generated great environmental differences, which might have triggered the diversification of species in the genus *Elymus*. Therefore, it can be speculated that the divergence time of the 44 *E. sibiricus* populations was significantly related to the uplift and climatic change of the QTP, which had given evidence that India–Asia collision and QTP uplift has progressed northeastward in a stepwise manner. Understanding LD patterns and mismatch distribution enhances the knowledge of the demographic processes within the population (Barría et al., 2013). The LD decay indicated Group I decay rate was low, which illustrated the strong natural selection. Natural selection has an important impact on shaping the genetic variation of a population, therefore promotes local adaptation (Kawecki and Ebert, 2004). Group I located in the northeast of the QTP, where human activities are frequent and the intensity of grassland utilization is high. It probably due to the increasing of human activities in the recent years (Group I is located in low altitude areas, and the country has made great efforts to develop tourism in the recent years, resulting in a substantial increase in the flow of people in this area). Thus, Group I was more selective, more adaptable, more diverse, and differentiated earlier. Group II and Group III possibly experienced expansion recently, but Group I likely experienced slight contraction. Group II experienced population contraction at 10 ka, the reason was the South-Tibetan river basin was affected by the Quaternary Glaciation. Regarding of the special geographical location with many land forms and micro-climates, it was be speculated that there had many small refuges of *E. sibiricus* on the QTP. Based on the analysis of genetic diversity and MaxEnt model results, theorizing that the QTP has multiple refuges in *E. sibiricus*, such as eastern Himalayas, Hengduan Mountains. Such area as the edge of the QTP (toward the northeastern, southeastern) might have an important role as an escape region.

The uplift of the plateau may lead to the change of habitat, and the adverse environment may lead to the contraction of plains in other areas (Liu et al., 2019). The uplift of the QTP has a great impact on China’s climate even the whole Asia (An, 2001), including the temperature decrease in some areas. Some cold-tolerant species with a wide range of habitats and vegetation zones certainly survived in multiple refugia on the QTP throughout glacial/inter-glacial periods. There were many Ice Age shelters on the edge of the QTP, which caused the alpine plants to retreat in the shelters during the Ice Age, and the population range of these plants began to expand during the inter-glacial or post-glacial warming. The MaxEnt model result of *E. sibiricus* also proved this historical process. The LGM is the period when the global temperature is the lowest, and it is also the coldest period recently. After the LGM, the temperature rose, the climate became warmer and humid, the suitable living area expanded, and the populations began to expand. These new climatic conditions may have been conducive to the expansion of populations of cold-resistant plants. During the glacial periods, most species experienced adverse climatic conditions in high-altitude mountain areas. Then contracted into refuges at low latitudes, and their ranges expanded again after the Ice Age, which leads to species divergence or secondary contact evolution (Hewitt, 2004; Nybom, 2004; Ohsawa and Ide, 2010). In the past century, the general trend of glacier change on the QTP retreated, and the marginal mountain area was more sensitive than the central area. The ecological niche modeling (ENM) results of grassland dominant species fit this pattern perfectly, partially mirroring the QTP historical change in grassland function. *Elymus sibiricus* most likely experienced slight population size change since the LGM. From the LIG to the LGM period, *E. sibiricus* exhibited significant range contraction. However, in the Mid-Holocene, the population range was expanded again. This is first report on the divergence time and demography of the most ecologically and economically important wheatgrass on the QTP high-cold grassland, which help us understand the grassland history.

Currently, the population distribution has decreased. The reason for this distribution pattern may be first that glaciers covered the QTP during the LGM, causing the populations retreat into the northeastern edge of the QTP and to the refuge. After the glacial period, the species of the Mid-Holocene re-expanded from the refuges. However, in the recent years, with the change of global climate and the increase of human activities, the suitable habitats for *E. sibiricus* were destroyed. It led to the decrease in *E. sibiricus* populations, especially in the northeast of QTP, but human activities were more intensive and the most significant climate warming. Additionally, according to the suitable distribution map, there was a refuge for *E. sibiricus* on the QTP during the glacial period, mainly distribute in the eastern Himalayas. As the climate warms, glaciers continue to retreat, accelerating the melting of permafrost. QTP climate warming causes regional and local climate change: soil exposure, severe desertification, grassland productivity decline. The future result of ENM showed the overall suitable area of *E. sibiricus* decreased compared to the Mid-Holocene, especially the moderate and high suitable areas. Therefore, the future of the QTP is not optimistic, it will keep warming trend, climate and ecological risk of disasters increased. It also funds that the distribution of *E. sibiricus* has shifted to the edge of the QTP. The change caused by climate warming, and would influence the distribution of *E. sibiricus* in the future. Combined with the genetic diversity and geographical distribution pattern of *E. sibiricus*, the regions with high genetic diversity were selected as the priority areas for protection and utilization, and scientific and accurate protection strategies were formulated. Thus, the reasonable and scientific preventions should be made to maintain the grassland function and protect the genetic germplasm of QTP *E. sibiricus* based on these evidences.

## Data Availability Statement

The datasets presented in this study can be found in online repositories. The names of the repository/repositories and accession number(s) can be found at: National Center for Biotechnology Information (NCBI) BioProject database under accession number PRJNA811778.

## Author Contributions

XY, YXG, and YZG designed the research. DL, CBZ, LY, LC, MY, WG, XL, JY, and SB provided individuals. MH and JZ performed the research and analyzed the data. MH wrote the manuscripts. XY, SS, and CJZ revised the manuscript. All authors contributed to the article and approved the submitted version.

## Conflict of Interest

The authors declare that the research was conducted in the absence of any commercial or financial relationships that could be construed as a potential conflict of interest.

## Publisher’s Note

All claims expressed in this article are solely those of the authors and do not necessarily represent those of their affiliated organizations, or those of the publisher, the editors and the reviewers. Any product that may be evaluated in this article, or claim that may be made by its manufacturer, is not guaranteed or endorsed by the publisher.

## Footnotes

1
http://timetree.org/

2
https://www.gbif.org/

3
http://www.worldclim.org/
