# Supplementary material for: Phylogeographical Pattern and Population Evolution History of Indigenous Elymus sibiricus L. on Qinghai-Tibetan Plateau
Source: Front Plant Sci. 2022 Jun 29;13:882601. doi: 10.3389/fpls.2022.882601 (PMC9277506; doi:10.3389/fpls.2022.882601)
Supplement: Supplementary file 1 [file Table_1.pdf]

Table S1 The specimen information about the sample number, latitude, longitude, and altitude for all of sampled populations.

Species ID	Sample numbers	Longitude	Latitude	Altitude
QH02	5	101.9703	36.4439	2600
QH03	5	101.8211	35.2069	3640
QH04	5	100.8253	35.2236	3340
QH06	5	101.4981	36.6608	2390
QH07	5	101.1708	36.5342	2930
QH08	5	97.3039	33.4103	4260
QH09	4	97.1928	33.6333	4310
QH10	5	97.3767	37.2814	2900
GS02	5	102.8178	35.2211	2550
GS03	5	102.6747	35.2065	2770
GS06	5	102.6788	34.4964	3010
GS07	5	102.6321	34.0944	3380
GS08	5	102.9164	34.9415	2960
GS09	5	103.1584	34.8369	3200
GS10	5	103.2473	34.6836	3160
GS11	5	103.5232	34.5673	2530
GS12	5	103.5859	34.4179	2540
GS14	5	103.1296	36.9576	2370
GS15	5	103.1483	36.9617	2430
SC02	5	102.62	31.98	3343
SC04	5	101.97	32.381	3334
SC07	5	103.248	33.641	2831
SC11	5	102.0877	32.7354	3571
SC13	5	101.1859	32.617	3061
SC14	5	100.9847	32.213	3243
SC15	5	100.7136	31.8703	3288
SC16	5	100.3466	29.8301	3972
SC17	4	101.445	29.4771	3311
XZ01	5	98.7231	29.7367	3530
XZ02	5	97.6845	29.8519	3909
XZ03	5	96.6705	29.9838	3776
XZ04	5	96.778	29.5407	4118
XZ05	5	92.7014	29.8798	3969
XZ06	5	92.935	30.0315	3646
XZ07	5	91.9016	29.7342	4025
XZ08	5	90.8238	29.7373	3790
XZ09	4	93.7356	31.7802	3950
XZ10	5	95.0346	31.6881	3827
XZ11	5	96.0164	31.216	3734
XZ12	5	96.6172	31.1882	3786
XZ13	4	97.2033	31.4772	3340
XZ14	5	97.2389	31.4808	3392
XZ15	5	97.9379	31.3246	3952
XZ16	5	98.1406	31.3762	3736
