# Supplementary material for: Phylogeographical Pattern and Population Evolution History of Indigenous Elymus sibiricus L. on Qinghai-Tibetan Plateau
Source: Front Plant Sci. 2022 Jun 29;13:882601. doi: 10.3389/fpls.2022.882601 (PMC9277506; doi:10.3389/fpls.2022.882601)
Supplement: Supplementary file 3 [file Table_3.pdf]

Table S3 Summary statistics for all populations of specific-locus amplified fragment sequencing (SLAF-seq) data processing

Sample ID	SLAF number	SNP number	Total depth	Average depth	Integrity	Heter ratio
QH02-1	401220	6775320	4691509	11.69	22.46%	0.94%
QH02-3	373915	5863096	4247334	11.36	19.44%	0.86%
QH02-5	395276	6685256	4594857	11.62	22.17%	0.95%
QH02-6	388850	7111761	4245417	10.92	23.58%	1.12%
QH02-7	390660	7165754	3562159	9.12	23.76%	1.22%
QH03-1	371209	6370227	3383887	9.12	21.12%	1.04%
QH03-3	281587	3090793	2158655	7.67	10.25%	0.66%
QH03-4	283268	2675039	3575549	12.62	8.87%	0.68%
QH03-6	294170	2801879	4102121	13.94	9.29%	0.96%
QH03-10	365246	5818670	3062146	8.38	19.29%	1.06%
QH04-2	232562	5228941	2789020	11.99	17.34%	6.54%
QH04-3	183133	2296896	2985779	16.3	7.62%	4.38%
QH04-4	232786	5375619	2720975	11.69	17.82%	6.93%
QH04-7	248567	6229736	3168951	12.75	20.66%	7.23%
QH04-8	225703	5571195	1734437	7.68	18.47%	7.07%
QH06-1	221557	4948881	2541473	11.47	16.41%	6.64%
QH06-2	233686	5519443	2825280	12.09	18.30%	6.98%
QH06-5	238877	5648633	2514619	10.53	18.73%	7.23%
QH06-7	244937	6006280	3321539	13.56	19.91%	7.34%
QH06-13	237868	4748560	3136161	13.18	15.74%	5.80%
QH07-1	227100	5649481	2301170	10.13	18.73%	7.25%
QH07-2	244390	5805449	2931872	12	19.25%	7.78%
QH07-3	229438	5656725	2268791	9.89	18.76%	7.26%
QH07-6	237493	5885552	2681697	11.29	19.51%	7.28%
QH07-10	222924	5019646	2209913	9.91	16.64%	6.37%
QH08-2	240111	5426627	3112229	12.96	17.99%	6.19%
QH08-3	234948	5999858	2492158	10.61	19.89%	6.98%
QH08-4	239280	6296722	2960912	12.37	20.88%	7.39%
QH08-5	222518	6339676	2191352	9.85	21.02%	7.72%
QH08-6	239633	6380895	3146765	13.13	21.16%	7.49%
QH09-1	387855	7221230	3500600	9.03	23.94%	1.23%
QH09-2	419900	8188782	3759442	8.95	27.15%	7.16%
QH09-3	253000	6680107	3523676	13.93	22.15%	7.53%
QH09-4	240324	6344665	2746958	11.43	21.04%	7.32%
QH10-1	233932	6676582	2322040	9.93	22.14%	7.79%
QH10-3	232756	5544524	2974854	12.78	18.38%	6.83%
QH10-4	233356	6582467	2622270	11.24	21.83%	7.62%
QH10-7	177783	2557876	1982297	11.15	8.48%	4.49%
QH10-9	198489	2898765	2993374	15.08	9.61%	4.85%
GS02-2	234679	4715179	3214570	13.7	15.63%	6.04%
GS02-4	240600	5347350	3358281	13.96	17.73%	6.71%
GS02-6	348367	6769970	2667070	7.66	22.45%	1.35%
GS02-8	401925	6482384	5167149	12.86	21.49%	1.05%
GS02-9	356778	6732965	2177756	6.1	22.32%	7.08%
GS03-2	385084	7152998	3127538	8.12	23.72%	1.17%
GS03-4	322782	3389797	3644201	11.29	11.24%	0.90%
GS03-5	332380	3638862	4155820	12.5	12.07%	0.72%
GS03-6	326409	3448903	3769540	11.55	11.44%	0.68%
GS03-7	316914	3233540	3822306	12.06	10.72%	0.73%
GS06-2	310537	3138723	3313810	10.67	10.41%	0.72%

GS06-3	319332	3280519	3695199	11.57	10.88%	0.69%
GS06-4	313183	3291043	3062065	9.78	10.91%	0.68%
GS06-6	338673	3684380	4848894	14.32	12.22%	0.71%
GS06-10	301730	2844778	3887273	12.88	9.43%	0.72%
GS07-1	392502	6655161	3942976	10.05	22.07%	1.14%
GS07-2	294990	2925580	3111523	10.55	9.70%	0.68%
GS07-3	287872	3136876	3437956	11.94	10.40%	0.73%
GS07-6	311648	3818618	3878339	12.44	12.66%	0.79%
GS07-12	320936	3745614	4626203	14.41	12.42%	0.78%
GS08-1	199733	2970213	2852286	14.28	9.85%	4.88%
GS08-6	251742	6846970	3633571	14.43	22.70%	7.69%
GS08-7	187299	2546563	3504115	18.71	8.44%	4.24%
GS08-8	200315	3086380	3163120	15.79	10.23%	4.50%
GS08-11	209979	3268340	3803153	18.11	10.84%	4.70%
GS09-3	129431	4171266	1798357	13.89	13.83%	8.08%
GS09-9	201962	2926698	3695968	18.3	9.70%	4.47%
GS09-10	179278	2568084	2585982	14.42	8.51%	4.29%
GS09-12	206061	3184518	3311161	16.07	10.56%	4.56%
GS09-13	207606	3118008	3640113	17.53	10.34%	4.60%
GS10-1	200361	3130798	3094477	15.44	10.38%	4.92%
GS10-8	148508	2179446	1479503	9.96	7.23%	4.00%
GS10-9	208358	3390822	4502459	21.61	11.24%	5.41%
GS10-10	238361	5337919	3036935	12.74	17.70%	6.13%
GS10-11	202150	3207095	3309277	16.37	10.63%	4.79%
GS11-1	224822	4209943	2367792	10.53	13.96%	5.19%
GS11-3	198126	2874737	3029420	15.29	9.53%	4.45%
GS11-8	361001	4952797	3163008	8.76	16.42%	0.75%
GS11-9	314664	3456635	4124538	13.11	11.46%	0.74%
GS11-10	201471	2948044	3223237	16	9.77%	4.51%
GS12-1	320796	3685407	4931702	15.37	12.22%	0.80%
GS12-2	359870	4556413	3235729	8.99	15.11%	0.73%
GS12-3	328931	3763642	4248916	12.92	12.48%	0.81%
GS12-4	307816	3000321	3264840	10.61	9.95%	0.72%
GS12-7	175266	2352860	2293742	13.09	7.80%	4.14%
GS14-3	204083	4382529	1500135	7.35	14.53%	5.63%
GS14-4	143079	1536178	1588667	11.1	5.09%	3.37%
GS14-7	152892	1807164	1544940	10.1	5.99%	3.61%
GS14-8	139448	1552998	1375780	9.87	5.15%	3.22%
GS14-9	205839	2844168	2989914	14.53	9.43%	4.60%
GS15-1	184422	2592261	2283097	12.38	8.60%	4.29%
GS15-6	223223	6460477	1783512	7.99	21.42%	8.47%
GS15-7	284644	5133811	2000234	7.03	17.02%	6.49%
GS15-8	213645	4776750	1849012	8.65	15.84%	6.12%
GS15-9	215385	4926301	2188065	10.16	16.33%	6.15%
XZ01-2	368843	7064784	2886326	7.83	23.42%	1.39%
XZ01-3	373106	7136511	2781550	7.46	23.66%	1.34%
XZ01-5	347020	6814163	2478272	7.14	22.59%	1.32%
XZ01-6	363874	7229608	2786112	7.66	23.97%	1.32%
XZ01-7	369941	7327986	2893685	7.82	24.30%	1.30%
XZ02-5	357111	7092049	2403052	6.73	23.51%	1.34%
XZ02-8	352042	6729001	2272521	6.46	22.31%	1.26%
XZ02-19	343742	6793711	2076579	6.04	22.53%	1.30%
XZ02-20	369285	5893542	3160285	8.56	19.54%	1.12%
XZ02-23	299829	6721888	1222387	4.08	22.29%	1.41%
XZ03-1	331992	6155978	2451249	7.38	20.41%	1.24%

XZ03-5	362456	7184824	2354582	6.5	23.82%	1.28%
XZ03-11	358846	7089843	1783943	4.97	23.51%	1.47%
XZ03-12	345362	6901722	2015776	5.84	22.88%	1.29%
XZ03-15	341487	6526721	1841331	5.39	21.64%	1.20%
XZ04-8	332065	6499908	1934052	5.82	21.55%	1.35%
XZ04-10	352131	6829297	2544772	7.23	22.64%	1.37%
XZ04-11	344429	4911185	2744028	7.97	16.28%	0.91%
XZ04-14	318856	5289983	1784762	5.6	17.54%	1.11%
XZ04-15	350827	7389695	2290244	6.53	24.50%	1.48%
XZ05-6	359882	6626907	2368696	6.58	21.97%	1.20%
XZ05-7	357623	7160254	2648562	7.41	23.74%	1.35%
XZ05-12	362344	6795928	3019821	8.33	22.53%	1.29%
XZ05-18	369526	7636098	2678046	7.25	25.32%	1.42%
XZ05-20	344009	6622917	2309614	6.71	21.96%	1.27%
XZ06-2	312067	7253094	1512788	4.85	24.05%	1.51%
XZ06-3	344364	7679805	1944661	5.65	25.46%	1.46%
XZ06-6	357335	7059568	2490599	6.97	23.41%	1.30%
XZ06-8	356456	6762488	2433211	6.83	22.42%	1.21%
XZ06-11	345905	6282901	2012756	5.82	20.83%	1.13%
XZ07-2	346412	6861304	2070830	5.98	22.75%	1.28%
XZ07-4	344177	6366517	2114114	6.14	21.11%	1.17%
XZ07-5	333206	6722109	1790008	5.37	22.29%	1.25%
XZ07-8	372872	7690718	2889742	7.75	25.50%	1.45%
XZ07-9	352339	6074766	2559494	7.26	20.14%	1.13%
XZ08-2	380225	7314267	3192156	8.4	24.25%	1.34%
XZ08-4	363731	7075519	2996932	8.24	23.46%	1.34%
XZ08-5	366841	6397742	2975446	8.11	21.21%	1.18%
XZ08-6	330971	4381811	2274510	6.87	14.53%	0.85%
XZ08-7	331792	4498795	2204955	6.65	14.92%	0.86%
XZ09-1	307427	3702575	2163627	7.04	12.28%	0.83%
XZ09-2	318447	4048462	2038707	6.4	13.42%	0.85%
XZ09-3	331874	4510557	2293046	6.91	14.96%	0.98%
XZ09-4	410447	8293718	3725429	9.08	27.50%	1.53%
XZ10-2	418573	9208754	3416545	8.16	30.53%	1.53%
XZ10-6	402114	8686785	3005754	7.47	28.80%	1.49%
XZ10-7	409036	8380031	3136883	7.67	27.79%	1.41%
XZ10-8	362702	7369271	1742185	4.8	24.43%	1.30%
XZ10-9	390264	8474989	2200286	5.64	28.10%	1.45%
XZ11-1	387994	7451511	2592708	6.68	24.71%	1.41%
XZ11-2	352690	4780948	3195754	9.06	15.85%	0.82%
XZ11-7	354905	4586320	3933100	11.08	15.21%	0.84%
XZ11-11	401930	8834011	2877150	7.16	29.29%	1.62%
XZ11-12	350235	4986881	2627698	7.5	16.53%	0.89%
XZ12-1	351461	5464554	2442913	6.95	18.12%	1.10%
XZ12-2	385124	6634985	3745939	9.73	22.00%	1.28%
XZ12-5	358088	4598437	4430917	12.37	15.25%	0.88%
XZ12-7	369928	4859425	3867516	10.45	16.11%	0.91%
XZ12-9	361579	4936906	4434869	12.27	16.37%	0.88%
XZ13-5	349845	4490808	3073843	8.79	14.89%	0.84%
XZ13-8	326932	6282213	1213964	3.71	20.83%	1.12%
XZ13-20	412819	9079433	2950816	7.15	30.10%	1.54%
XZ13-21	414264	8326176	2994650	7.23	27.61%	1.36%
XZ14-1	348355	4479021	2653415	7.62	14.85%	0.74%
XZ14-3	353882	4405898	3717671	10.51	14.61%	0.77%
XZ14-10	344639	4179882	3303112	9.58	13.86%	0.78%

XZ14-12	354158	4749141	3005775	8.49	15.75%	0.77%
XZ14-13	414501	8349951	3092927	7.46	27.69%	1.39%
XZ15-1	356309	4443946	3645187	10.23	14.73%	0.77%
XZ15-4	408384	8658680	3013021	7.38	28.71%	1.42%
XZ15-5	413841	9038416	3274398	7.91	29.97%	1.48%
XZ15-6	402605	8406463	2547320	6.33	27.87%	1.36%
XZ15-7	353178	4642127	3224870	9.13	15.39%	0.77%
XZ16-1	410065	8626094	3141669	7.66	28.60%	1.87%
XZ16-4	365457	5010258	4195743	11.48	16.61%	0.79%
XZ16-7	352943	4579953	5005156	14.18	15.19%	0.82%
XZ16-10	415634	8741695	3570622	8.59	28.98%	1.57%
XZ16-11	418260	9134666	3553775	8.5	30.29%	1.66%
SC02-1	388737	4993126	3112081	8.01	16.56%	0.94%
SC02-3	386929	5587412	5334434	13.79	18.53%	0.80%
SC02-5	383489	5884749	3894421	10.16	19.51%	1.04%
SC02-6	418864	8467548	3325325	7.94	28.08%	1.46%
SC02-9	430494	9034964	4774335	11.09	29.96%	1.53%
SC04-2	399730	7841882	2859629	7.15	26.00%	1.28%
SC04-5	412383	8720434	4002678	9.71	28.91%	1.49%
SC04-12	364869	4942990	3922148	10.75	16.39%	0.78%
SC04-13	406846	7991444	3201812	7.87	26.50%	1.36%
SC04-17	407838	7639522	3538721	8.68	25.33%	1.31%
SC07-1	407966	7708661	3714955	9.11	25.56%	1.44%
SC07-13	414367	8313256	3913914	9.45	27.56%	1.38%
SC07-14	401792	8573802	2978168	7.41	28.43%	1.45%
SC07-17	399295	7371715	2801261	7.02	24.44%	1.23%
SC07-22	391402	7449858	2604696	6.65	24.70%	1.25%
SC11-1	381088	5152559	4908428	12.88	17.08%	0.86%
SC11-2	406185	7561047	3615272	8.9	25.07%	1.31%
SC11-5	393248	7754584	2691204	6.84	25.71%	1.36%
SC11-4	364559	4655628	4381535	12.02	15.44%	0.76%
SC11-7	361989	4846547	3732556	10.31	16.07%	0.79%
SC13-3	391349	6896112	2877988	7.35	22.87%	1.20%
SC13-8	380360	7254883	2280678	6	24.05%	1.28%
SC13-9	405678	7352581	3105246	7.65	24.38%	1.26%
SC13-12	392715	7177748	2946504	7.5	23.80%	1.25%
SC13-13	397179	7692095	2995364	7.54	25.50%	1.33%
SC14-6	399657	7825686	3145003	7.87	25.95%	1.31%
SC14-7	401957	7382807	3023141	7.52	24.48%	1.23%
SC14-9	401565	7469517	3140954	7.82	24.77%	1.29%
SC14-11	354364	4714436	3780235	10.67	15.63%	0.79%
SC14-14	375095	5101500	4712549	12.56	16.91%	0.81%
SC15-4	390864	5884240	4753381	12.16	19.51%	0.94%
SC15-6	353528	4141069	3608987	10.21	13.73%	0.75%
SC15-7	340832	3888686	4095523	12.02	12.89%	0.74%
SC15-10	371112	4231941	3980068	10.72	14.03%	4.59%
SC15-11	411715	7405042	4791320	11.64	24.55%	1.13%
SC16-1	411783	8006263	4386328	10.65	26.55%	1.36%
SC16-2	303620	3754251	1803048	5.94	12.45%	0.87%
SC16-3	314806	3959650	1906738	6.06	13.13%	0.89%
SC16-6	353161	4801362	2362686	6.69	15.92%	0.98%
SC16-7	336955	3615551	4335915	12.87	11.99%	0.77%
SC17-1	377086	5924040	2524915	6.7	19.64%	1.12%
SC17-2	367776	6144866	2298403	6.25	20.37%	1.10%
SC17-3	367216	5187325	2711193	7.38	17.20%	1.05%

SC17-4	381757	5605176	4487984	11.76	18.58%	0.82%
