# Supplementary figures and images for: Phylogeographical Pattern and Population Evolution History of Indigenous Elymus sibiricus L. on Qinghai-Tibetan Plateau
Source: Front Plant Sci. 2022 Jun 29;13:882601. doi: 10.3389/fpls.2022.882601 (PMC9277506; doi:10.3389/fpls.2022.882601)

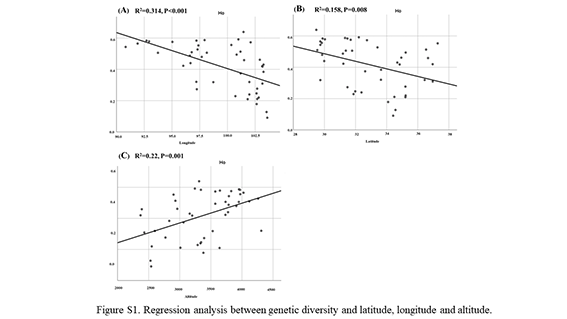

Supplement: Supplementary file 4 [file Image_1.tif]

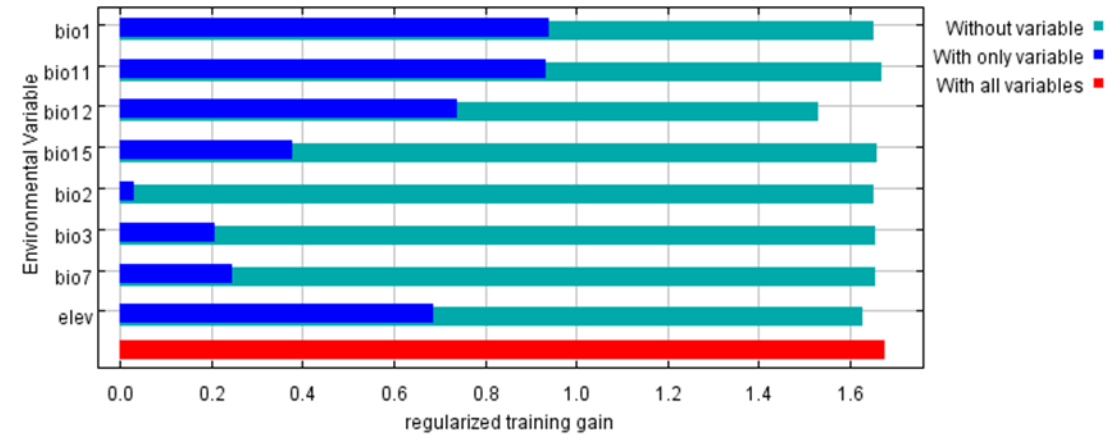

Figure S3. Key Environmental variables based on Jackknife method

Supplement: Supplementary file 6 [file Image_3.pdf]
