# Supplementary material for: Phylogeographical Pattern and Population Evolution History of Indigenous Elymus sibiricus L. on Qinghai-Tibetan Plateau
Source: Front Plant Sci. 2022 Jun 29;13:882601. doi: 10.3389/fpls.2022.882601 (PMC9277506; doi:10.3389/fpls.2022.882601)
Supplement: Supplementary file 5 [file Image_2.pdf]

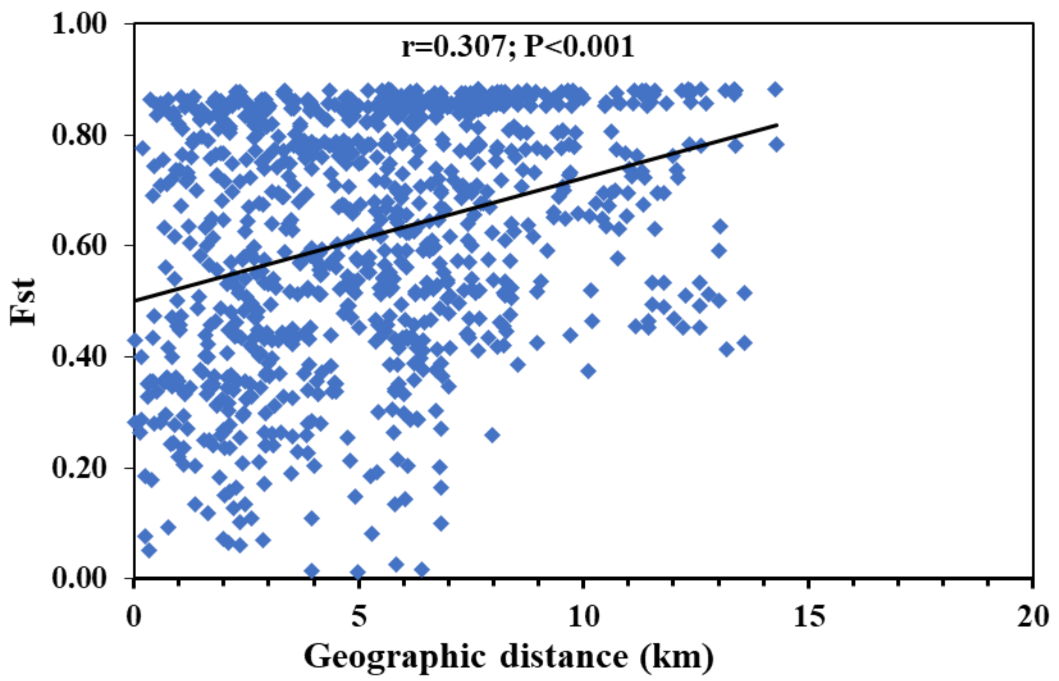

Figure S2. Isolation-by distance between all population pairs where Fst was regressed over the geographic distance
